# Human milk oligosaccharides induce acute yet reversible compositional changes in the gut microbiota of conventional mice linked to a reduction of butyrate levels

**DOI:** 10.1093/femsml/uqac006

**Published:** 2022-05-18

**Authors:** Andrea Qvortrup Holst, Harshitha Jois, Martin Frederik Laursen, Morten O A Sommer, Tine Rask Licht, Martin Iain Bahl

**Affiliations:** National Food Institute, Technical University of Denmark, DK-2800 Kgs. Lyngby, Denmark; National Food Institute, Technical University of Denmark, DK-2800 Kgs. Lyngby, Denmark; Glycom / DSM, DK-2970 Hørsholm, Denmark; National Food Institute, Technical University of Denmark, DK-2800 Kgs. Lyngby, Denmark; Novo Nordisk Foundation Center for Biosustainability, Technical University of Denmark, DK 2800 Kgs. Lyngby, Denmark; National Food Institute, Technical University of Denmark, DK-2800 Kgs. Lyngby, Denmark; National Food Institute, Technical University of Denmark, DK-2800 Kgs. Lyngby, Denmark

**Keywords:** HMO, bacteroides, prebiotic, microbiota, butyrate, microbiome

## Abstract

Human Milk Oligosaccharides (HMOs) are glycans with prebiotic properties known to drive microbial selection in the infant gut, which in turn influences immune development and future health. Bifidobacteria are specialized in HMO degradation and frequently dominate the gut microbiota of breastfed infants. However, some species of *Bacteroidaceae* also degrade HMOs, which may prompt selection also of these species in the gut microbiota. To investigate to what extent specific HMOs affect the abundance of naturally occurring *Bacteroidaceae* species in a complex mammalian gut environment, we conducted a study in 40 female NMRI mice administered three structurally different HMOs, namely 6’sialyllactose (6'SL, *n* = 8), 3-fucosyllactose (3FL, *n* = 16), and Lacto-N-Tetraose (LNT, *n* = 8), through drinking water (5%). Compared to a control group receiving unsupplemented drinking water (*n* = 8), supplementation with each of the HMOs significantly increased both the absolute and relative abundance of *Bacteroidaceae* species in faecal samples and affected the overall microbial composition analyzed by 16s rRNA amplicon sequencing. The compositional differences were mainly attributed to an increase in the relative abundance of the genus *Phocaeicola* (formerly *Bacteroides*) and a concomitant decrease of the genus *Lacrimispora* (formerly *Clostridium* XIVa cluster). During a 1-week washout period performed specifically for the 3FL group, this effect was reversed. Short-chain fatty acid analysis of faecal water revealed a decrease in acetate, butyrate and isobutyrate levels in animals supplemented with 3FL, which may reflect the observed decrease in the *Lacrimispora* genus. This study highlights HMO-driven *Bacteroidaceae* selection in the gut environment, which may cause a reduction of butyrate-producing clostridia.

## Teaser

The study investigates effects of human milk oligosaccharides on the intestinal bacterial community in a rodent model and highlights changes affecting faecal butyrate concentrations.

## Introduction

The newborn gut microbiota is typically characterized by a low bacterial diversity and dominated by facultative anaerobic bacteria, reflecting the oxygenated state of the gut at birth (Bäckhed et al. 2015, Ferretti et al. 2018). The microbiota then undergoes large, but mostly conserved structural changes on a successional path towards a well-established microbiota achieved at about 3 years of age (Stewart et al. 2018, Laursen et al. 2021). Several different external factors influence these structural changes including early life exposure to antibiotics (Nobel et al. 2015), while the most important natural influencer is human breast milk (Stewart et al. 2018). Breast milk contains structurally diverse Human Milk Oligosaccharides (HMOs, 5–25 g/l) with about 200 different natural forms categorized into fucosylated, sialylated, and nonfucosylated neutral structures (Bode 2012, Chen 2015). The HMOs have no direct nutritional value for the infant, as humans lack the enzymatic capabilities to hydrolyse these compounds (Brand-Miller et al. 1998, Gnoth et al. 2000). However, low levels of HMOs have been detected in the blood of the breastfed infants suggesting they may confer systemic health benefits (Goehring et al. 2014). However, the majority of HMOs reach the colon where they selectively promote growth of certain bacteria in the developing gut microbiota and, thus have a prebiotic capacity (Bode 2015). Specifically, HMOs have been found to be highly important in establishing and maintaining a community rich in infant-associated bifidobacteria such as *Bifidobacterium longum* ssp. *infantis* with relatively low overall bacterial diversity (Laursen et al. 2021). In accordance with this, a recent study in 12 infants sampled densely during the first 2 years (*n* = 1048), points to the cessation of breastfeeding as the key to the switch from an infant-like bifidobacteria-dominated community towards an adult-like community dominated by Clostridiales and Bacteroidales (Tsukuda et al. 2021). Notably, the study also demonstrated that the increase in Clostridiales is associated with a concurrent increase in faecal butyrate, which is typically almost absent in very early life. Some bacterial species within the *Bacteroidaceae* family are also able to utilize HMOs as a carbon source (Yu et al. 2013, Salli et al. 2021). Specifically, the species *Phocaeicola vulgatus*, formerly *Bacteroides vulgatus* (García-López et al. 2019), has in several independent studies been shown to obtain high *in vitro* growth on the major fucosylated and sialylated HMOs namely 3-fucosyllactose (3FL), 2′-fucosyllactose (2′FL), lactodifucosyllactose (LDFL), 3′-sialyllactose (3′SL), and 6'SL (Yu et al. 2013, Salli et al. 2021).

The potential competition for HMOs between bifidobacteria and Bacteroidales has been investigated both *in vitro* and *in vivo* and is proposed to be based on differences in specificity for uptake of the HMO (Marcobal et al. 2011). *Bacteroides* species are generalists and use a mucin-glycan degradation pathway for HMO utilization, while infant-type bifidobacteria are specialists and grow efficiently on HMOs but not on mucin glycans. The specificity of bifidobacteria might give them the selective advantage observed when inoculated in a 1:1 competition with *Bacteroides* species using a HMO supplemented germ-free mouse model (Marcobal et al. 2011). Nonetheless, in experiments with animals colonized with a complex adult microbiota, *Bacteroidaceae* respond positively to HMO supplementation (Pruss et al. 2020). However, in this study animals were consuming a microbiota-accessible carbohydrate deficient diet, and it is unknown if similar *Bacteroidaceae* enriching effects of HMO supplementation would apply if animals are consuming a complex fibre rich diet.

It is possible that expansion of Bacteroides species observed during the complementary feeding period (the transition from exclusive breastfeeding to family foods) could be driven by the combined presence of HMOs and introduction of more complex carbohydrates and fibres reflecting their metabolic capacities (Laursen et al. 2017, Stewart et al. 2018). In addition, in the adult setting, with limited abundance of HMO-degrading *Bifidobacterium* species, *Bacteroidaceae* may enrich during prebiotic HMO supplementation. Indeed, potential benefits of HMOs as a prebiotic supplement besides infant nutrition have been considered (Elison et al. 2016, Fonvig et al. 2021, Iribarren et al. 2021). A recent trial in healthy adults showed that oral supplementation with 2'-O-fucosyllactose (2'FL), lacto-N-neotetraose (LNnT), or a mix of the two up to 20 g/day for 2 weeks (*n* = 10/group) was well-tolerated and linked to an increase in the relative abundance of Actinobacteria and specifically *Bifidobacterium* coupled to a reduction in Firmicutes and Proteobacteria (Elison et al. 2016). The relevance of bifidobacteria as a marker for a healthy intestinal community in adults is, however, not well-established (Schnorr et al. 2014), so other HMO induced microbiota changes with potential health effects should also be considered. In the present study, we sought to investigate modulatory effects of oral HMO supplementation, including 3FL, Lacto-N-Tetraose (LNT), and 6’sialyllactose (6’SL) on bacterial community composition, short-chain fatty acids concentrations and colonic gene expression in a NMRI mouse model lacking infant-type bifidobacteria. The chosen compounds represent abundant and commercial available fucosylated, basic neutral and sialylated HMOs found in human breast milk.

## Materials and methods

### HMO drinking water solutions

3FL, LNT, and 6’SL in powder form was obtained from Glycom A/S with a purity above 92% (3FL = 92.4%; LNT = 95.5%; and 6'SL = 98.8%). HMOs were separately dissolved in autoclaved water, sterile filtered (0.45 µm) and adjusted to a concentration of 50 g/l (5% w/v) in a total volume of 600 ml. From these solutions water was transferred to individual drinking water bottles. Animals in the CTR group received unsupplemented autoclaved water. Drinking bottles were refilled when necessary. In a previous pilot trial the drinking water intake of NMRI mice (same age and gender) was found to be approximately 5 ml per day with no difference observed between pure (autoclaved) and 3FL (5%) supplemented drinking water. The daily intake of HMOs was thus calculated to be approximately 0.25 g/animal/day.

### Design of the animal study

**Figure 1.**
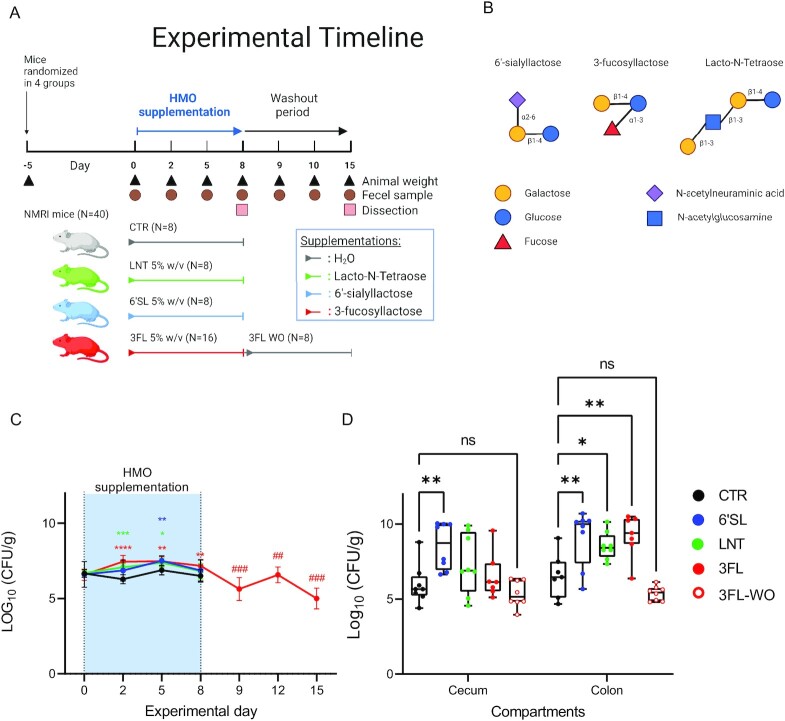
Experimental design and *Bacteroidaceae* culturing data in NMRI mice. (A) The experimental study design of the animal trial is shown indicating period of HMO supplementation and sampling times. (B) chemical structure of the three different HMOs, 6'SL, 3FL, and LNT included as study substrates. (C) Enumeration of CFUs from faecal samples obtained before, during, and after the HMO supplementation period is shown as mean values with error bars indicating standard deviations. The period of HMO supplementation in drinking water is highlighted as a shaded box. (D) Box-plots showing CFU counts of samples obtained from the cecum and colon at the end of the HMO supplementation period and after the 1-week washout period for the 3FL-WO group. Individual values are shown with whiskers highlighting minimum and maximum values. *P*-values were obtained by mixed-effects analysis followed by Dunnett's multiple comparisons tests between CTR and all HMO supplementation groups individually with *P < .05, **P < .01, ***P < .001, and ****P < .0001 or repeated-measures ANOVA followed by Dunnett's multiple comparisons tests comparing washout period time points to Day 8 CFU counts in the 3FL group with ^##^*P* < .01 and ^###^*P* < .001.

A total of 40 conventional NMRI, 6-weeks-old, female mice were obtained from Taconic and kept in a Scantainer under a 12 h light:dark cycle at a temperature of 22 ± 1° and relative humidity of 55 ± 5%. The mice were fed ad libitum Altromin 1314 chow (Brogaarden ApS, Lynge, Denmark) and autoclaved water in drinking bottles. A total of 4 days after arrival, on experimental Day 5 the mice were pseudo-randomized according to weight in four experimental groups; three groups of eight mice and one group of 16 mice and housed in cages of two (Fig. 1A). On experimental Day 0, before starting the treatment, the mice were weighed and faecal samples collected. The drinking bottles from each cage were exchanged with bottles containing clean autoclaved water, which for the treatment groups were supplemented with 50 g/l LNT, 50 g/l 6'SL, or 50 g/l 3FL, respectively. Animals in the CTR were provided nonsupplemented autoclaved water. On experimental Days 2, 5, and 8, the mice were weighed and faecal samples were collected directly from the animals. On Day 8, all mice of the CTR, 6'SL, and LNT groups (*n* = 8/group) and half of the mice in the 3FL group (*n* = 8) were anesthetized (hypnorm/midazolam) for collecting portal vein blood before being euthanization by cervical dislocation. The remaining half of the 3FL group mice (*n* = 8) continued into the washout period where the drinking bottles were exchanged with clean bottles containing autoclaved water without the supplementation. The 3FL group was selected based on data from a pilot study. On experimental Days 9, 12, and 15, the remaining mice were weighed and faecal samples collected. On Day 15, the eight remaining mice of the washout group were euthanized as described. All faecal samples were kept at room temperature in 2-ml tubes until processing immediately after the sampling. The mouse experiment was approved by the Danish Animal Experiments Inspectorate (license no. 2020–15–0201–00484 C nr.: C-1) and was overseen by the National Food Institute's in-house Animal Welfare Committee for animal care and use.

### Dissection

The intestines were dissected to obtain the tissue samples from mid-colon stored in RNA*later* (Invitrogen^TM^) for gene expression analysis and pellets from distal-colon for culturing and SCFA analysis. Colon samples were kept on ice until processing immediately after the dissection.

### Plating, enumeration, and isolation of *Bacteroidaceae* strains

Selective plating of Bacteroidaceae was performed on Brucella laked blood agar prepared from Brucella Agar with Hemin and Vitamin K1 (B2926 Sigma-Aldrich) supplemented with 50 ml/l filter-sterilized defibrinated sheep blood (no. 8545090 E&O Laboratories), 50 mg/l kanamycin, and 10 mg/l vancomycin (BrLa+Kan+Van agar). On the day of use, the plates were prewarmed at room temperature for approximately 4 h before plating appropriate dilutions of intestinal content. Faecal samples obtained from individual animals or intestinal content obtained upon dissection were weighed and then homogenized in 500 µl phosphate-buffered saline (PBS) by the pipet tip followed by 10 s vortexing on maximum speed. The resulting faecal/intestinal solution was 10-fold serially diluted in 96-well plates using a multichannel pipet with automatic mixing steps between rows. From each dilution, 5 µl was spot plated onto BrLa+Kan+Van agar plates and incubated for 2 days under anaerobic conditions at 37°C before enumeration. Enumeration of Bacteroidaceae was performed as CFU counts of spots with 5–20 visible isolates, summed between triplicates of each dilution, and multiplied by the dilution factor. Additionally, colonies from 12 randomly selected plates from dissections on Day 8 (HMO-supplemented animals) were further used for strain isolation and taxonomic identification. Two colonies from each of the 12 plates were restreaked on fresh BrLa+Kan+Van agar and grown for additional 2 days as stated above. Purified colonies were aseptically inoculated into a 1-ml cryo-vial containing Luria–Bertani (LB) broth and 15% glycerol, vortexed and stored at −80°C until further use.

### Isolate identification

The putative *Bacteroidaceae* isolates obtained from colon and faecal samples and intestinal content on the dissection day were plated from glycerol stocks on fresh BrLa+Kan+Van agar and grown for 2 days under anaerobic conditions at 37°C. Genomic DNA from the 22 isolates that regrew was extracted by use of the DNeasy UltraClean Microbial DNA Isolation kit (no. 12224–50, Qiagen) according to the manufacturer's protocol. Mechanical lysis of bacterial cells was performed at 30 cycles/s for 10 min on a bead beater MM300 (Retsch VWR). DNA concentrations were measured by the Qubit dsDNA HF kit (Q33266, Invitrogen) and samples diluted with nuclease-free water (W4502 Sigma-Aldrich) to a concentration of 5 ng/µl. The 16S rRNA gene sequences were amplified in a 50-µl PCR reactions containing 10 µl 5X Phusion™ HF-Buffer, 1 µL dNTPs (10 mM of each oligo), 1 µM universal forward primer 27F (AGA-GTT-TGA-TCM-TGG-CTC-AG), 1 µM universal reverse primer 1492R (TAC-GGY-TAC-CTT-GTT-ACG-ACT-T), 1 µl template DNA (5 ng/µl), and 0.5 µL Phusion™ High-Fidelity DNA polymerase (F-530 Thermo Scientific). Reaction conditions were as follows: Initial denature 98°C for 30 s, 35 cycles of 98°C for 15 s, 61°C for 15 s, 72°C for 60 s, and lastly 72°C for 5 min before cooling to 4°C. The PCR products were purification by use of the MinElute PCR purification kit (no. 28004 Qiagen) and diluted to 20–80 ng/µl with nuclease-free water. Each purified PCR product (5 µl) was mixed with 5 µl forward primer 27F (5 pmol/µl) and shipped to Eurofins facility (Eurofins Genomic Sequencing GMBH 51105 Köln, Germany) for Sanger-sequencing.

### Handling of fecal samples for gut microbiota and SCFA analyses

After the initial dilution of faecal samples described in the above section, the samples were centrifuged at 16 000 × *g* for 10 min at 4°C and 500 µl supernatant saved in 1.5-ml tubes at −20°C for later SCFA analysis. The pellet was stored at −20°C until bacterial DNA extractions. Bacterial DNA extraction was conducted by use of the DNeasy PowerLyzer PowerSoil Kit (no. 12855–50 Qiagen) essentially according to manufactures recommendations. Mechanical lysis of bacterial cells was performed at 30 cycles/s for 10 min on a bead beater MM300 (Retsch VWR). DNA concentrations were measured by the Qubit dsDNA HF kit (Q33266, Invitrogen) and adjusted to 5 ng/µl in nuclease-free water (W4502 Sigma-Aldrich).

### Gut microbiota analysis

Microbiota profiling was performed essentially as previously described (Laursen et al. 2021). Briefly, the V3 region of the 16s rRNA gene in extracted community DNA was PCR amplified by using a universal forward primer with a unique 10–12 basepair barcode for each sample (PBU 5´-A-adapter-TCAG-barcode-CCTACGGGAGGCAGCAG-3´) and a universal reverse primer (PBR 5´-trP1-adapter-ATTACCGCGGCTGCTGG-3´) in 20 µl reactions containing 4 µl 5X Phusion™ HF-Buffer, 0.4 µl dNTPs (10 mM of each oligo), 1 µM forward primer, 1 µM reverse primer, 1 µl template DNA (5 ng/µl) and 0.2 µl Phusion™ High-Fidelity DNA polymerase (F-530 Thermo Scientific). Reaction conditions were as follows: Initial denature 98°C for 30 s, 24 cycles of 98°C for 15 s, 72°C for 30 s, and lastly 72°C for 5 min before cooling to 4°C. The PCR products were purified by the HighPrep™ PCR clean-up system (AC-60500 Magbio) according to the manufacturer's protocol. The resulting DNA concentrations were determined by Qubit HS assay and libraries constructed with mixing equimolar amounts of each PCR product. Partial 16S rRNA gene sequencing was performed on an Ion S5™ System (ThermoFisher Scientific) using OneTouch 2 Ion5: 520/530 kit—OT2 400 bp and an Ion 520 Chip.

### Sequence data handling

Raw sequence data was initially quality checked and sequencing depth deemed satisfactory. The sequences were imported into CLC genomic workbench (v8.5, CLCbio, Qiagen) as FASTQ files, demultiplexed and trimmed using defaults settings. Reads below 125 bp and above 180 bp were discarded. The trimmed sequences were exported to Rstudio (version 4.0.5; Team RStudio 2015) and the Divisive Amplicon Denoising Algorithm 2 (DADA2) pipeline (Callahan et al. 2016) was used (standard settings, except pool = true and adjustments recommended for Ion Torrent reads, namely HOMOPOLYMER_GAP_PENALTY = -1, BAND_SIZE = 32, were implemented in the dada() function) to generate Amplicon Sequence Variants (ASVs), which were taxonomically classified using the Ribosomal Database Project database (rdp_train_set_18; Wang et al. 2007). The ASV taxonomic classification table and the ASV sequences and counts per sample were imported into Quantitative Insights Into Microbial Ecology 2 software (QIIME 2 Core–2020.11; Bolyen et al. 2019) and data sorted to contain only taxa of bacterial origin with very rare reads sorted out by setting a minimum frequency of 100 across all samples. Alpha and beta diversity metrics were calculated by the function ‘Diversity Core-metrics-phylogenetic’ based on a rooted phylogenetic tree. When performing diversity analysis each sample was rarefied to 11 000 reads to obtain even sampling depths. For beta diversity, when applicable, the data were sorted according to either experimental day (when performing ANOSIM or ANCOM analysis) or to taxonomic level when performing beta diversity analysis on a subset of the data. Relative abundance calculations were based on nonrarefied reads.

### Alignment and generation of a phylogenetic tree for 16S rRNA genes from isolates and microbial profiling

The 16s rRNA gene sequences obtained by Sanger sequencing of each isolate were quality assessed by CLC Main Workbench and trimmed to obtain only high-quality nucleotide reads. The sequences were searched against the National Center for Biotechnology Information (NCBI) Nucleotide database by the NCBI BlastN tools and the top match was applied as the putative taxonomic classification (Altschul et al. 1990). From the Ribosomal Database Project (rdp.cme.msu.edu) the 16S rRNA gene sequence from a total of 11 type strains representing both the *Bacteroides* genus and the *Phocaeicola* genus were downloaded and exported into CLC Main Workbench. Furthermore, sequences of ASVs taxonomically classified as either *Bacteroides* or *Phocaeicola* were sorted and the relative abundance of each ASV was summed across all samples. ASVs with a relative abundance sum above 1% were also imported into CLC Main Workbench using the ASV number and genus name as the identifier of the sequence. All sequences were trimmed to the same length (V3 region) before performing a multiple alignment and creating a phylogenetic tree with the algorithm ‘Neighbour-Joining’, the distance measure of ‘Jukes-Cantor’ and the bootstrap setting of 100 replicates.

### Short-chain fatty acids analysis of faecal waters

Faecal water samples obtained on the dissection day were prepared by thawing faecal supernatants at room temperature followed by centrifugation at 16 000 × *g* at 4°C for 5 min. The supernatants were then filtered through Costar SpinX centrifuge filters 0.22 µm (CLS8160 Sigma-Aldrich) at 15 000 × *g* for 5 min until clear. The filters were removed from the columns and the solutions were immediately couriered to MS-Omics (Vedbæk, DK-2950, Denmark) where they were stored at −80°C until analysis as follows. Samples were acidified using hydrochloride acid, and deuterium-labelled internal standards were added. All samples were analyzed in a randomized order. Analysis was performed using a high polarity column (Zebron™ ZB-FFAP, GC Cap. Column 30 m × 0.25 mm × 0.25 µm) installed in a GC (7890B, Agilent) coupled with a quadrupole detector (5977B, Agilent). The system was controlled by ChemStation (Agilent). Raw data were converted to netCDF format using Chemstation (Agilent) before the data was imported and processed in Matlab R2014b (Mathworks, Inc.) using the PARADISe software described by Johnsen et al (2017).

### RNA isolation from colonic tissue

Tissue samples from the colon were collected at dissection and stored in RNAlater (Sigma-Aldrich) at −80°C until further analysis. Approximately 30 mg of the tissue sample was used to purify total RNA. Samples were homogenized using a tissue-lyzer (QIAGEN Tissue lyser II) followed by total RNA purification (no. 74106, Qiagen), using Qiagen RNeasy with on-column DNase digestion using Qiagen RNase free DNase kit (no. 79254, Qiagen). cDNA synthesis was performed using omniscript c-DNA synthesis kit (no. 205113, Qiagen), Random primer mix (no. S1330S, Bio Nordica), and Anti RNAse (no. AM2694, Invitrogen) according to manufacturer's protocol.

### RT- qPCR, gene expression analysis

The real‐time quantitative PCR was carried out using Roche light cycler Real‐Time PCR System (Roche) and threshold cycle values were calculated by light cycler software (Roche). Reactions were performed in triplicates in 384‐well PCR plates (Thermo Scientific). The total volume in each well was 10 µl, containing 3 µl diluted cDNA (1:24), 5 µl Taqman Fast Advanced Master Mix (no. 4444963, ThermoFisher Scientific), and 0.5 µl Taqman Gene expression Assay primer/probe mix (ThermoFisher Scientific). Gene assays used were Occludin (Mm00500912_m1), Tight junction protein 1 (Tjp 1 Mm00493699_m1), Tumor necrosis factor-a (*TNF*-α Mm00443258_m1), and Mucin 2 (Muc2 Mm01276696_m1), chosen to represent tight junction, mucin and an inflammatory cytokine all potentially affected by modification of the gut microbiota and/or exposure to HMOs. Glyceraldehyde 3-phosphate dehydrogenase (GAPDH Mm99999915_g1) and Beta-Actin (*β*-*actin* Mm00607939_s1) were used as reference genes. Thermal cycling conditions for the reaction were as follows: 1 cycle at 50°C for 2 min, 1 cycle at 95°C for 20 s, 45 cycles at 95°C for 3 s, and 60°C for 30 s. The relative gene expression of the target genes was calculated using the 2^−ΔΔCt^ method and normalized with the housekeeping genes *GAPDH and β*-*actin*.

### Statistics

GraphPad Prism Software was used for statistical analysis unless otherwise stated. *t*-tests were applied for testing differences in means between two groups when data were normally distributed while a Mann–Whitney test was applied when data were not normally distributed. One- or two-way ANOVA tests or mixed-effects analysis were used when appropriate with multiple comparisons performed by Dunnett's *post-hoc* test. Alternatively, Kruskal–Wallis nonparametric tests with Dunn's multiple comparisons test were used. Correlation analysis was performed by Spearman rank's analysis. Microbial profiling data obtained by 16S rRNA gene sequencing were analyzed with QIIME2, employing ANOSIM for testing differences in the community between groups (beta diversity) and the ANCOM test for assessing differently abundant taxa between groups and sampling times using default settings (Clarke 1993, Mandal et al. 2015).

## Results

### HMO supplementation increased the absolute abundance of *Bacteroidaceae* family bacteria enumerated by culturing

The conventional NMRI mice used in this study tolerated supplementation of 5% HMO in their drinking water well and no differences in weight between groups were observed (Figure S1A, Supporting Information). From experimental Days 0 to 15 *Bacteroidaceae* colony forming units (CFU) in fecal samples were enumerated (Fig. 1A). No differences were found between the groups on Day 0 before HMO supplementation commenced (Fig. 1C, *P =* .28, Kruskal–Wallis test). Supplementation with HMOs resulted in significant changes in *Bacteroidaceae* levels (Fig. 1C, *P =* .0006, mixed-effects analysis). After 2 days of supplementation of HMOs in drinking water (5% 6’SL, LNT, or 3FL), higher counts of *Bacteriodaceae* were observed in the LNT and 3FL groups compared to the control group (CTR) while all three treatment groups were significantly higher on Day 5 (Fig. 1C*, P* < .05, Dunnett's multiple comparisons test). On Day 8 most of the animals were terminated, yet a washout group was maintained in the 3FL group (3FL-WO, *n* = 8) and followed for an additional 7 days without HMO supplementation, which resulted in a significant decrease in *Bacteroidaceae* (Fig. 1C*, P* < .0001, one-way ANOVA) compared to Day 8. Already 1 day after removing 3FL from the drinking water (Day 9), a significant decrease in *Bacteroidaceae* was observed as compared to Day 8 (*P =* .0004, Dunnett's multiple comparisons test). Counts of *Bacteroidaceae* remained decreased in 3FL-WO mice compared to Day 8 during the remainder of the washout period (Fig. 1C).

*Bacteroidaceae* CFUs were enumerated in the cecum and colon content after euthanization on Day 8 (*n* = 8 per group) and Day 15 from the 3FL-WO (*n* = 8). We found significant differences between both compartments (*P =* .0014), with overall higher counts in colon and between treatment groups (*P* < .0001) by mixed-effects analysis. In both cecum and colon, the 6’SL group had significantly higher levels of *Bacteroidaceae* compared to the CTR group, while all three HMO treatment groups resulted in higher *Bacteroidaceae* counts in the colon. (Fig. 1D*, P* < .05, Dunnett's multiple comparison test). After the washout period, levels of *Bacteroidaceae* were not significantly different from the CTR group in any of the compartments (Fig. 1D).

### Cultured *Bacteroidaceae* belonged to several genera and matched the Amplicon Sequence Variants identified by 16S rRNA gene sequencing

**Figure 2.**
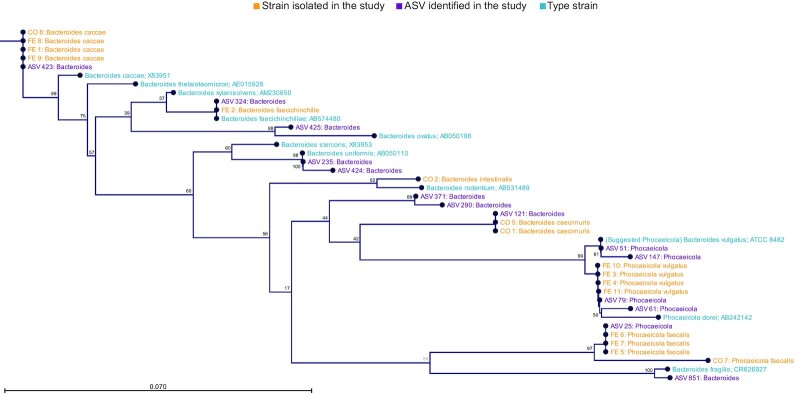
Phylogenetic tree based on cultured strains, ASVs identified by 16S rRNA gene sequencing and reference type strains. The tree was generated by the ‘Neighbour-Joining’ method using the ‘Jukes-Cantor’ distance measures with bootstrap values shown (100 replicates). The bar shows phylogenetic distance.

A total of 21 bacterial isolates from Day 8 faecal and colon samples from several different animals were picked randomly from different animals, excluding the CTR group. Identification by partial 16s rRNA gene Sanger sequencing confirmed that colonies mostly belonged to the *Bacteroidaceae* family with strains representing several different *Bacteroides* and *Phocaeicola* species (Table S1, Supporting Information). A total of four of the 21 isolates did not belong to the *Bacteroidaceae* family. Alignment of the V3 regions of the 16S rRNA gene sequences obtained from isolated colonies with the most prevalent *Bacteroidaceae* ASVs identified by 16S rRNA gene profiling (Table S2, Supporting Information) showed an overall good match between the two methods (Fig. 2). The generated phylogenetic tree including also type strains revealed distinct clades for the genus *Bacteroides* and the genus *Phocaeicola*, underlining the taxonomic differences between these recently separated genera. The only exception was the species *Bacteroides fragilis*, which seemed more closely related to *Phocaeicola faecalis* based on this dataset. The genus *Phocaeicola* itself also appeared to be further divided into two distinct clades represented by *P. vulgatus* and *P. fragilis* respectively, with phylogenetic distances as large as those found between *Bacteroides* and *Phocaeicola* genera, indicating substantial phylogenetic variation between species within the *Phocaeicola* genus (Fig. 2).

### HMO supplementation affected microbial composition dependent on the specific compound

**Figure 3.**
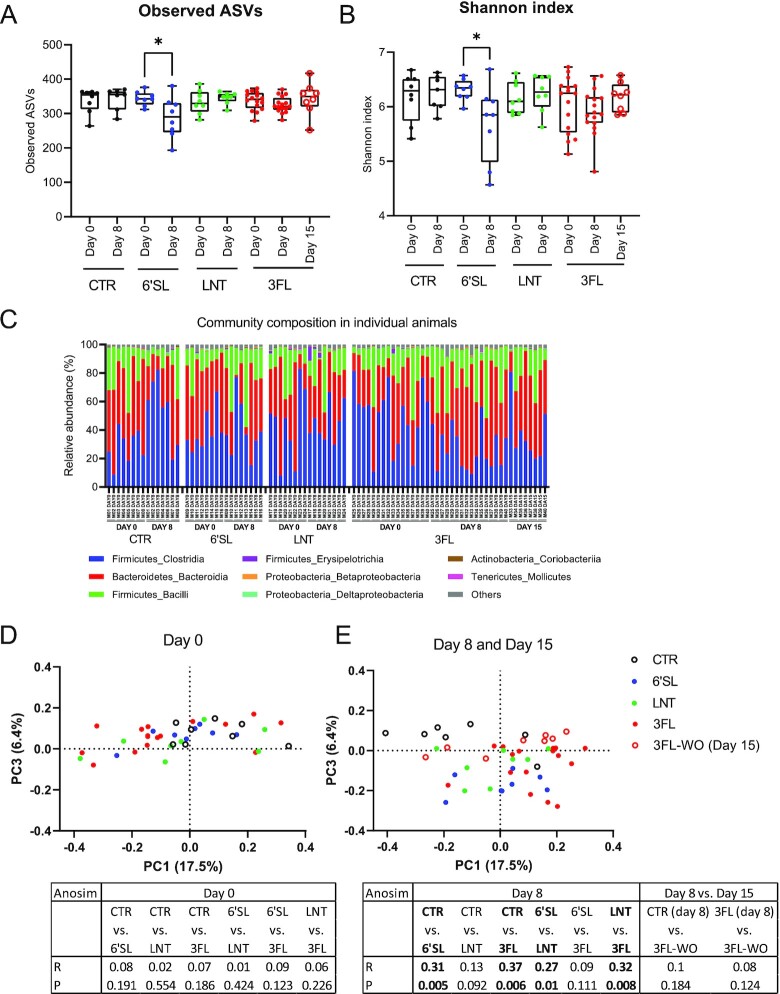
Effects of HMO supplementation on bacterial diversity and community composition. (A) Boxplots of total number of observed ASVs and (B) Shannon index based on 16S rRNA amplicon sequencing. Statistical significance between time-points was evaluated by paired *t*-tests within groups with *P < .05. (C) Profiling of bacterial composition in faecal samples obtained from individual animals indicates relative abundance at the class level. Grey lines indicate cocaging of animals. (D) Principal coordinate analysis based on Bray–Curtis dissimilarity on Day 0 and (E) Day 8/Day 15 coloured by group. The table shows results of pairwise comparisons performed by ANOSIM tests indicating R and *P-*values with significant differences highlighted in bold (*P* < .05).

Microbial profiling by 16S rRNA gene sequencing before and after HMO supplementation showed a marked reduction of both richness (observed number of ASVs) and Shannon diversity in animals that had received 6’SL (Fig. 3A and B*, P* < .05, paired *t*-test), while no differences in alpha diversity were observed in the LNT and 3FL groups. The relative abundance of bacterial classes in faecal samples for individual animals at Days 0, 8, and 15 showed no clear indications of specific changes for HMO supplemented animals at this level, which could be explained by the Bacteroidia class predominantly consisting of *Muribaculaceae* (approximately 70% on average) while the *Bacteroidaceae* only constituted approximately 6% of this class (Fig. 3C). Principal coordinate analysis based on Bray–Curtis dissimilarity matrices clearly indicated differences in faecal microbial composition between treatment groups and CTR after HMO supplementation (Day 8), which CTR samples clustering separately from the other samples (high PC3-value). No differences were observed before supplementation (Day 0), indicating a specific effect of the HMOs on the microbiota (Fig. 3D and E). Analysis of similarities (ANOSIM) revealed that the faecal microbiota in animals receiving 6’SL and 3FL were significantly different from that in the CTR group on Day 8, while the LNT group did not differ from the CTR group (Fig. 3D and E). Interestingly, the 3FL-WO samples obtained on Day 15 after the washout period showed a reversion from the 3FL sample cluster towards the CTR sample cluster, with no significant difference found by ANOSIM analyses between CTR Day 8 and 3FL-WO groups (Fig. 3E, *P =* .184, ANOSIM test). We did not observe notable cage-effects in the study (Fig. 3C; Figure S1F, Supporting Information).

### The genus *Phocaeicola* increased in relative abundance during HMO supplementation while the genus *Lacrimispora* decreased

**Figure 4.**
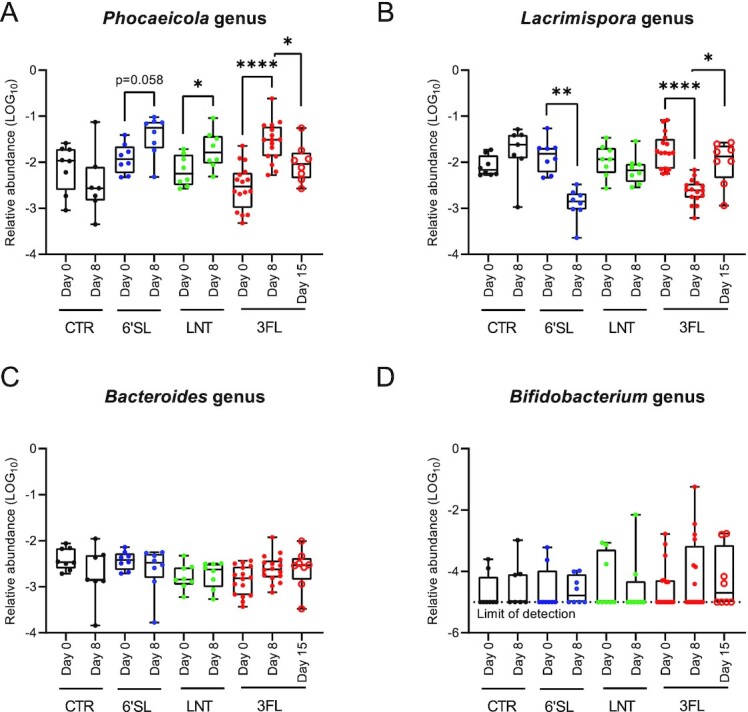
Effects of HMO supplementation on relative abundance of specific bacterial genera. (A) Boxplots showing the relative abundance of *Phocaeicola*, (B) *Lacrimispora*, (C) *Bacteroides*, and (D) *Bifidobacterium* based on 16S rRNA amplicon sequencing. Statistical significance between time-points was evaluated by paired *t*-tests within groups. *P < .05, **P < .01, ****P < .0001.

To investigate which bacterial genera contributed to the observed changes in microbial composition during HMO supplementation, a statistical analysis of the compositional changes between Days 0 and 8 (ANCOM test) was performed at the genus level for all four treatment groups separately. Using this stringent test we found one genus, *Phocaeicola* (formerly *Bacteroides*) significantly increase (3FL group) and one genus, *Lacrimispora* significantly decrease (6SL group) from Days 0 to 8, although same trends were observed for both 3FL and 6'SL treatment groups (Figure S2, Supporting Information). Focusing specifically on the genera found to be differently abundant by the ANCOM analysis, paired *t*-tests were applied to compare the relative abundances of *Phocaeicola* and *Lacrimispora*, respectively, within each group between Days 0 and 8. This showed that all three HMOs caused an expansion of *Phocaeicola* and a concomitant decrease in *Lacrimispora*, particularly pronounced in the 3FL group (Fig. 4A and B). In accordance with culturing data, the washout period resulted in a relative abundance of *Phocaeicola* on Day 15 that was significantly reduced compared to Day 8 (Fig. 4A). Additionally, *Lacrimispora* was significantly increased on Day 15 compared to Day 8 (Fig. 4B). No HMO-induced changes in levels of *Bacteroides* (not including *Phocaeicola*) nor *Bifidobacterium* between Days 0 and 8 were found in any of the treatment groups (Fig. 4C and D). Strong positive and negative correlations were found between the calculated PC3 coordinates (Fig. 3E) and the relative abundance of *Lacrimispora* (Rho = 0.60*, P < *.0001, Spearman's rank correlation), and *Phocaeicola* (Rho = −0.69*, P < *.0001, Spearman's rank correlation), respectively indicating that these genera were driving the observed differences in the PCoA plot. Another interesting finding from the ANCOM analysis was that the abundance of the genus *Faecalibacterium* was significantly lower on Day 15 in the 3FL-WO group compared to both Days 0 and 8, with the genera being below detection level in all samples on Day 15 (Figures S1E, S2E, and S2F, Supporting Information).

### Supplementation with 3FL-reduced faecal concentrations of acetate, butyrate, and isobutyrate

**Figure 5.**
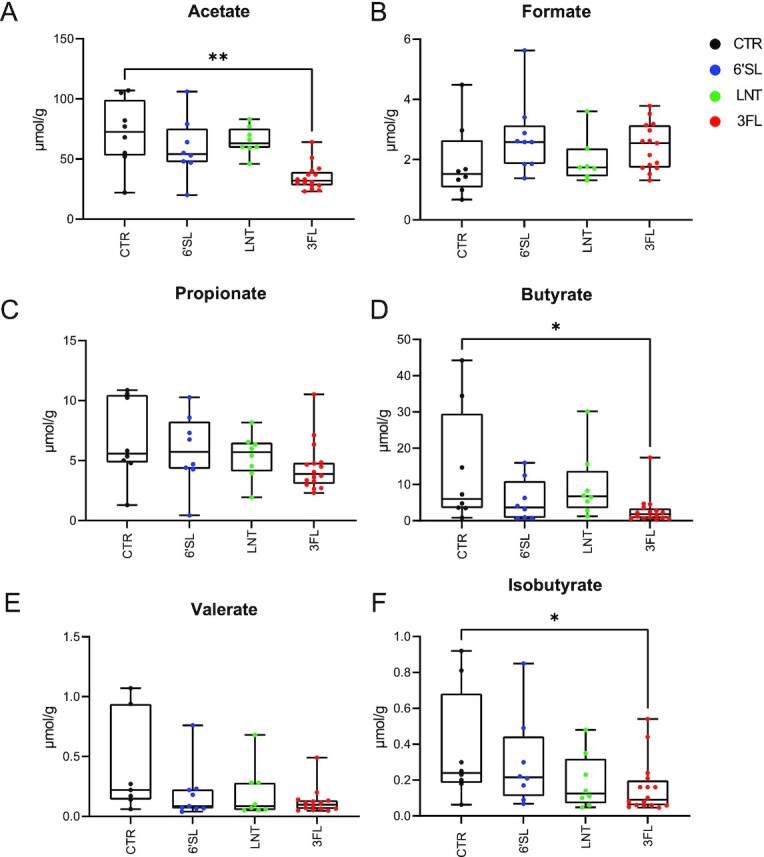
Effects of HMO supplementation on faecal short-chain fatty acid concentrations. (A) Boxplots showing the concentration of acetate, (B) formate, (C) propionate, (D) butyrate, (E) valerate, and (F) isobutyrate. Values below detection level (LOD) are shown as the LOD. Statistical significance between groups was evaluated by Kruskal–Wallis tests followed by Dunn's multiple comparisons tests comparing to the CTR group. *P < .05 and **P < .01.

The concentrations of short-chain fatty acids measured in faecal samples of all animals on Day 8 revealed significant differences in concentrations of acetate, butyrate, and isobutyrate for HMO-treated animals compared to the CTR group (Fig. 5*, P* < .05, Kruskal–Wallis tests). Specifically lower concentrations of acetate (*P =* .0027, Dunn's multiple comparisons test), butyrate (*P =* .025, Dunn's multiple comparisons test), and isobutyrate (*P =* .048, Dunn's multiple comparisons test) were found in the 3FL group compared to CTR. No other HMO-induced differences in SCFA levels were found.

### Colonic occludin expression lowered during and after HMO supplementation

**Figure 6.**
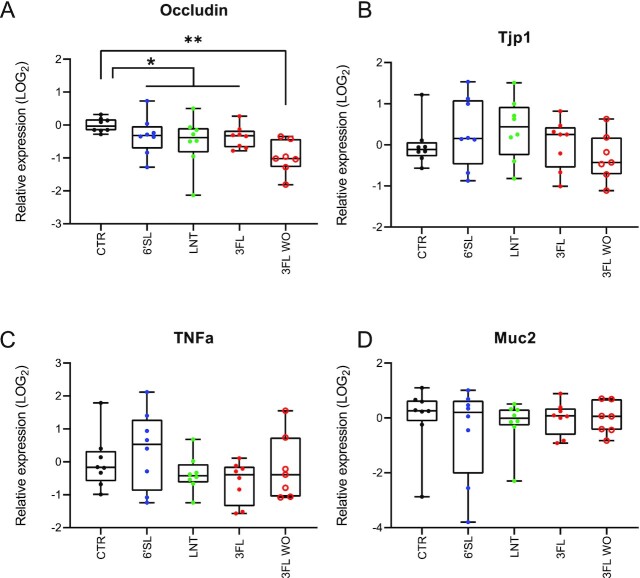
Effects of HMO supplementation on colonic tissue gene expression. (A) Boxplots showing the relative gene expression of occludin, (B) Tjp1, (C) TNFα, and (D) Muc2. Statistical significance between all groups was evaluated by Kruskal–Wallis tests followed by Dunn's multiple comparisons tests comparing to the CTR group or Mann–Whitney test between the 6'SL, LNT, and 3FL groups aggregated vs. the CTR group. *P < .05 and **P < .01.

We found no effects of HMO supplementation on colonic gene expression levels of Tjp1, TNFα, and Muc2 compared to the CTR group on Day 8 (Fig. 6A–D). However, a significantly lower gene expression level of occludin was observed in the 3FL-WO group on Day 15 compared to the CTR group on Day 8 (*P =* .0005, Dunn's multiple comparisons test). A comparison between the CTR group and all three treatment groups aggregated on Day 8 also revealed slightly, but significantly lower levels of occludin gene expression compared to the CTR group (Fig. 6A, *P =* .012, Mann–Whitney test).

### HMO-induced expansion of *Phocaeicola* and reductions in *Lacrimispora* is associated with reduced faecal acetate and butyrate levels

**Figure 7.**
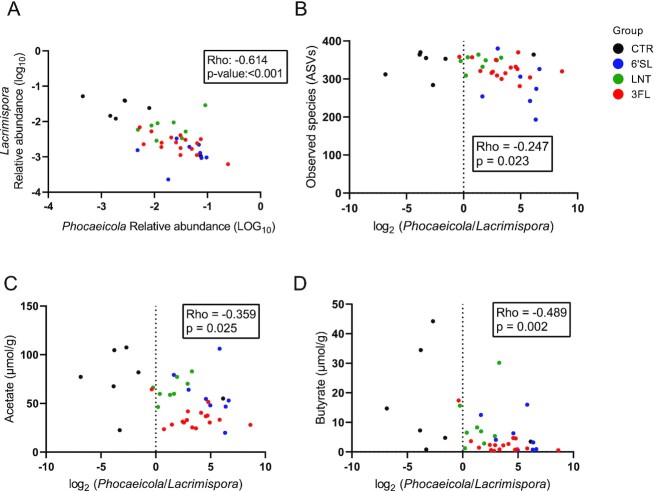
Correlations between affected bacterial genera, alpha diversity and short-chain fatty acids in faecal samples. (A) Scatter dot plots of *Lacrimispora* vs. *Phocaeicola* relative abundances, (B) *Phocaeicola*/*Lacrimispora* (log_2_) ratio vs. number of observed ASVs, (C) *Phocaeicola*/*Lacrimispora* (log_2_) ratio vs. concentration of acetate and (D) *Phocaeicola*/*Lacrimispora* (log_2_) ratio vs. concentration of butyrate. Associations were assessed by Spearman's rank correlation analysis with rho and *P*-values indicated.

A highly significant negative correlation was found between the relative abundance of *Phocaeicola* and *Lacrimispora* (Fig. 7A, Rho = −0.614*, P* < .001, Spearman's rank correlation). Focusing on the the negatively correlated *Phocaeicola* to *Lacrimispora* we found that the ratio between these two genera correlated negatively with the number of observed species (Fig. 7B, Rho = −0.247, *P =* .023, Spearman's rank correlation), indicating general effects on the community composition. We further found negative correlations between the *Phocaeicola* to *Lacrimispora* ratio and levels of both faecal acetate (Fig. 7C, rho = −0.359, *P =* .025, Spearman's rank correlation) and butyrate (Fig. 7D, rho = −0.489, *P =* .002, Spearman's rank correlation) but not isobutyrate (rho = −0.11, *P =* .51). Clustering of samples according to specific HMO treatment group (indicated by different colours) were notable in the dot-plots (Fig. 7A–D).

## Discussion

The investigated HMOs were well-tolerated in mice at the given dosage, which is consistent with previous studies in humans (Elison et al. 2016) at comparable dosages (Nair and Jacob 2016). To investigate the effects of HMO supplementation specifically on the culturable members of the *Bacteroidaceae* family we used Brucella lacked blood agar supplemented with kanamycin and vancomycin as previously described (Sheppard et al. 1990). This growth media proved to be a reliable method for tracking acute changes in the *Bacteroidaceae* abundance during HMO supplementation (Fig. 1C) and strain isolation (Fig. 2; Table S1, Supporting Information). Interestingly, the positive correlation between *Bacteroidaceae* CFU/g and *Phocaeicola* relative abundance on Day 8 (Figure S1D, Supporting Information) was not present on Day 0 (Figure S1C, Supporting Information), indicating that the strains enumerated on Day 0 were not predominantly *Phocaeicola* strains but probably a broader collection of *Bacteroidaceae* species, while the positive correlation observed on Day 8 indicated that mainly *Phocaeicola* strains were cultured at this time point and, thus selected for by HMOs. Enumeration of CFUs from cecum and colon samples likewise showed a general increase in *Bacteroidaceae* in HMO-supplemented animals compared to control animals, which were more pronounced in colon samples than in the cecum samples (Fig. 1D), which could reflect a higher selective effect in the colon, possibly enhanced by mucus glycan metabolism (Donaldson et al. 2016, Patnode et al. 2019). A positive selection for *Bacteroidaceae* species during administration of selective carbohydrates has been reported in several previous studies, although *Bacteroidaceae* species may be outcompeted by specialist HMO degrading *Bifidobacterium* species when coinoculated in a germ-free mouse model during HMO supplementation (Marcobal et al. 2011). Also, the mucin-derived O-glycans vs. HMO availability may affect growth competition between *Bifidobacterium* and *Bacteroidaceae* (Pruss et al. 2020). As very low abundances of bifidobacteria were detected in the mice applied in the present study, and these most probably were not adapted to HMO degradation, the *Bacteroidaceae* strains were likely not challenged by direct competition with bifidobacterial species (Fig. 4D). This study underlined the general ability of *Bacteroidaceae* species to readily respond to changes in carbohydrate availability and exploit a new nutritional niche especially in the absence of specialist HMO degrading bifidobacteria.

In the present study, HMOs were found to affect the faecal microbial composition after 8 days supplementation period (Fig. 3) with effects observed already on Day 2 (Fig. 1C). The negatively correlated genera *Phocaeicola* and *Lacrimispora* were found to be the main drivers of the observed difference of the HMO-supplemented animals in all three treatment groups. The observed expansion of *Phocaeicola* is consistent with previous *in vitro* studies (Yu et al. 2013, Salli et al. 2021). The phylum Bacteroidetes has recently undergone taxonomic reclassification based on a large genome-scale survey (García-López et al. 2019). Here, it was proposed to reclassify some former *Bacteroides* species into the genus *Phocaeicola* including *B. dorei* and *B. vulgatus* while most other prevalent species remain in the *Bacteroides* genus including *B. fragilis* and *B. thetaiotaomicron*. Interestingly, this study showed that HMO supplementation did not affect the now constricted *Bacteroides* genera (Fig. 4C).

Analysis of SCFA levels revealed a significant decrease of butyrate and acetate in the 3FL-supplemented group (Fig. 5A and D) compared to the CTR group, which could be linked to the observed reduction in *Lacrimispora* spp. The family of *Lachnospiraceae* (formerly designated as part of *Clostridium* cluster XIVa) are among the main producers of butyrate in the gut (Van Den Abbeele et al. 2013, Vacca et al. 2020). The *Clostridium sphenoides* group has recently been reclassified as the genus *Lacrimispora* under the *Lachnospiraceae* family and most species from this genus are confirmed to have the genetic capacity to synthesize butyrate through the acetyl-coenzyme A (CoA) pathway (Vital et al. 2014, Haas and Blanchard 2019). In line with this, the relative abundance of *Lacrimispora* was significantly and positively correlated with faecal levels of butyrate (Figure S3D, Supporting Information). *Lacrimispora* also correlated positively with faecal acetate levels collectively indicating a role of *Lacrimispora* in faecal SCFA levels (Figure S3B, Supporting Information), while no correlation with isobutyrate was found. Whether the observed HMO induced decrease in *Lacrimispora* was caused by a change in the gut environment (e.g. pH) or by competition with *Phocaeicola* remains unresolved. *In vitro* studies have shown that lowering pH levels by 1 unit in anaerobic continuous cultures curtails the dominating bacterial population of *Bacteroidaceae* species relative to that of *Clostridia* species such as the family of *Lachnospiraceae* and that the shift is correlated to a metabolic response resulting in a large increase in butyrate production (Walker et al. 2005). The butyrate producing *Faecalibacterium* was present in most faecal samples on Days 0 and 8 but was, surprisingly, absent (below level of detection), in samples on Day 15 (Figures S1E, S2E, and S2F, Supporting Information). We speculate that *Faecalibacterium* may also be negatively affected by 3FL-induced changes in the community composition possibly due to the significantly decreased levels of acetate (substrate for butyrate production) observed on Day 8 (Duncan et al. 2002, Wrzosek et al. 2013), but further studies are needed.

The observed reduction of occludin gene expression (Fig. 6A) linked to barrier function through tight junction stability (Cummins 2012, Panwar et al. 2021, Pérez-Reytor et al. 2021) was most pronounced in the 3FL washout group (Day 15) compared to CTR (Day 8) but also significant when all HMO groups were combined (Day 8). The cause of this reduction is difficult to determine based on available data, but could be speculated to be linked to the observed reduction in faecal butyrate levels (Fig. 5D), although no significant correlation was found.

The HMOs 3FL, LNT, and 6’SL are considered safe for human consumption as a novel food supplementation (Turck et al. 2019, 2020, 2021) and several clinical trials have reported no adverse effects upon HMO-supplementation of adults and children 6–12 years of age (Elison et al. 2016, Palsson et al. 2020, Fonvig et al. 2021, Iribarren et al. 2021). None of the clinical studies conducted in either adults or children have analyzed changes in microbial short-chain fatty acids as a marker of overall microbial activity and gut health (Pérez-Reytor et al. 2021). However, our findings suggest that this may be relevant. A limitation of the study was that combinations of HMOs were not addressed in the experimental design.

In conclusion, this study demonstrates an acute yet reversible HMO-induced increase in the human-relevant *Phocaeicola* (formerly *Bacteroides*) concurrent with a reduction in butyrate-producing *Lacrimispora* in the context of a complex, mature microbiota of conventional mice. This was linked to a decrease in faecal butyrate levels especially following supplementation with the fucosylated HMO 3FL. The reported results additionally emphasize the importance of including other effects than bifidogenicity when evaluating the effects of HMOs in a complex adult-like microbiota.

## Data availability

The 16S rRNA gene sequence data are deposited in the NCBI Sequence Read Archive with the accession number PRJNA787049.

## Authors' contribution

A.Q.H., T.R.L., M.F.L., and M.I.B. conceived and designed the study. A.Q.H. and H.J. performed the experimental work. A.Q.H., H.J., and M.I.B. analyzed and interpreted the data. A.Q.H. drafted the manuscript. All authors made substantial intellectual contributions, revised the manuscript, and approved the final version of the manuscript.

## Supplementary Material

uqac006_Supplemental_Files

## References

[bib1] Altschul SF , Gish W, Miller W et al. Basic local alignment search tool. J Mol Biol. 1990;215:403–10. 2231712 10.1016/S0022-2836(05)80360-2

[bib2] Bäckhed F , Roswall J, Peng Y et al. Dynamics and stabilization of the human gut microbiome during the first year of life. Cell Host Microbe. 2015;17:690–703. 25974306 10.1016/j.chom.2015.04.004

[bib3] Bode L. Human milk oligosaccharides: every baby needs a sugar mama. Glycobiology. 2012;22:1147–62. 22513036 10.1093/glycob/cws074 PMC3406618

[bib4] Bode L. The functional biology of human milk oligosaccharides. Early Hum Dev. 2015;91:619–22. 26375354 10.1016/j.earlhumdev.2015.09.001

[bib5] Bolyen E , Rideout JR, Dillon MR et al. Reproducible, interactive, scalable and extensible microbiome data science using QIIME 2. Nat Biotechnol. 2019;37:852–7. 31341288 10.1038/s41587-019-0209-9 PMC7015180

[bib6] Brand-Miller JC , McVeagh P, McNeil Y et al. Digestion of human milk oligosaccharides by healthy infants evaluated by the lactulose hydrogen breath test. J Pediatr. 1998;133:95–8. 9672518 10.1016/s0022-3476(98)70185-4

[bib7] Callahan BJ , McMurdie PJ, Rosen MJ et al. DADA2: high-resolution sample inference from Illumina amplicon data. Nat Methods. 2016;13:581–3. 27214047 10.1038/nmeth.3869 PMC4927377

[bib8] Chen X. Human milk oligosaccharides (HMOS): structure, function, and enzyme-catalyzed synthesis. Adv Carbohydr Chem Biochem. 2015;72:113–90. 26613816 10.1016/bs.accb.2015.08.002 PMC9235823

[bib9] Clarke KR. Non-parametric multivariate analyses of changes in community structure. Austral Ecol. 1993;18:117–43.

[bib10] Cummins PM. Occludin: one protein, many forms. Mol Cell Biol. 2012;32:242–50. 22083955 10.1128/MCB.06029-11 PMC3255790

[bib11] Donaldson GP , Lee SM, Mazmanian SK. Gut biogeography of the bacterial microbiota. Nat Rev Microbiol. 2016;14:20–32. 26499895 10.1038/nrmicro3552 PMC4837114

[bib12] Duncan SH , Hold GL, Harmsen HJM et al. Growth requirements and fermentation products of *Fusobacterium prausnitzii*, and a proposal to reclassify it as *Faecalibacterium prausnitzii* gen. nov., comb. nov. Int J Syst Evol Microbiol. 2002;52:2141–6. 12508881 10.1099/00207713-52-6-2141

[bib13] Elison E , Vigsnaes LK, Rindom Krogsgaard L et al. Oral supplementation of healthy adults with 2′-O-fucosyllactose and lacto-N-neotetraose is well tolerated and shifts the intestinal microbiota. Br J Nutr. 2016;116:1356–68. 27719686 10.1017/S0007114516003354 PMC5082288

[bib14] Ferretti P , Pasolli E, Tett A et al. Mother-to-Infant microbial transmission from different body sites shapes the developing infant gut microbiome. Cell Host Microbe. 2018;24:133–145. 30001516 10.1016/j.chom.2018.06.005 PMC6716579

[bib15] Fonvig CE , Amundsen ID, Vigsnæs LK et al. Human milk oligosaccharides modulate fecal microbiota and are safe for use in children with overweight: a randomized controlled trial. J Pediatr Gastroenterol Nutr. 2021;73:408–14. 34139746 10.1097/MPG.0000000000003205

[bib16] García-López M , Meier-Kolthoff JP, Tindall BJ et al. Analysis of 1,000 type-strain genomes improves taxonomic classification of bacteroidetes. Front Microbiol. 2019;10:2083. 31608019 10.3389/fmicb.2019.02083 PMC6767994

[bib17] Gnoth MJ , Kunz C, Kinne-Saffran E et al. Human milk oligosaccharides are minimally digested in vitro. J Nutr. 2000;130:3014–20. 11110861 10.1093/jn/130.12.3014

[bib18] Goehring KC , Kennedy AD, Prieto PA et al. Direct evidence for the presence of human milk oligosaccharides in the circulation of breastfed infants. PLoS ONE. 2014;9:e101692. 24999728 10.1371/journal.pone.0101692 PMC4085000

[bib19] Haas KN , Blanchard JL. Reclassification of the *Clostridium clostridioforme* and *Clostridium sphenoides* clades as *Enterocloster* gen. nov. and *Lacrimispora* gen. nov., including reclassification of 15 taxa. Int J Syst Evol Microbiol. 2020;70:23–34. 31782700 10.1099/ijsem.0.003698

[bib20] Iribarren C , Magnusson MK, Vigsnæs LK et al. The effects of human milk oligosaccharides on gut microbiota, metabolite profiles and host mucosal response in patients with irritable bowel syndrome. Nutrients. 2021;13:3836. 34836092 10.3390/nu13113836 PMC8622683

[bib21] Johnsen LG , Skou PB, Khakimov B et al. Gas chromatography – mass spectrometry data processing made easy. J Chromatogr A. 2017;1503:57–64. 28499599 10.1016/j.chroma.2017.04.052

[bib22] Laursen MF , Bahl MI, Licht TR. Settlers of our inner surface-factors shaping the gut microbiota from birth to toddlerhood. FEMS Microbiol Rev. 2021;45:fuab001. 33428723 10.1093/femsre/fuab001 PMC8371275

[bib23] Laursen MF , Bahl MI, Michaelsen KF et al. First foods and gut microbes. Front Microbiol. 2017;8:356. 28321211 10.3389/fmicb.2017.00356 PMC5337510

[bib24] Laursen MF , Pekmez CT, Larsson MW et al. Maternal milk microbiota and oligosaccharides contribute to the infant gut microbiota assembly. ISME Commun. 2021;1:1–13. 10.1038/s43705-021-00021-3 PMC9723702 36737495

[bib25] Mandal S , Van Treuren W, White RA et al. Analysis of composition of microbiomes: a novel method for studying microbial composition. Microb Ecol Heal Dis. 2015;26:27663. 10.3402/mehd.v26.27663 PMC4450248 26028277

[bib26] Marcobal A , Barboza M, Sonnenburg ED et al. Bacteroides in the infant gut consume milk oligosaccharides via mucus-utilization pathways. Cell Host Microbe. 2011;10:507–14. 22036470 10.1016/j.chom.2011.10.007 PMC3227561

[bib27] Nair A , Jacob S. A simple practice guide for dose conversion between animals and human. J Basic Clin Pharm. 2016;7:27. 27057123 10.4103/0976-0105.177703 PMC4804402

[bib28] Nobel YR , Cox LM, Kirigin FF et al. Metabolic and metagenomic outcomes from early-life pulsed antibiotic treatment. Nat Commun. 2015;6:ncomms8486. 10.1038/ncomms8486 PMC4491183 26123276

[bib29] Palsson OS , Peery A, Seitzberg D et al. Human milk oligosaccharides support normal bowel function and improve symptoms of irritable bowel syndrome: a multicenter, open-label trial. Clin Transl Gastroenterol. 2020;11:e00276. 33512807 10.14309/ctg.0000000000000276 PMC7721220

[bib30] Panwar S , Sharma S, Tripathi P. Role of barrier integrity and dysfunctions in maintaining the healthy gut and their health outcomes. Front Physiol. 2021;12:1573. 10.3389/fphys.2021.715611 PMC8497706 34630140

[bib31] Patnode ML , Beller ZW, Han ND et al. Interspecies competition impacts targeted manipulation of human gut bacteria by fiber-derived glycans. Cell. 2019;179:59–73. 31539500 10.1016/j.cell.2019.08.011 PMC6760872

[bib32] Pérez-Reytor D , Puebla C, Karahanian E et al. Use of short-chain fatty acids for the recovery of the intestinal epithelial barrier affected by bacterial toxins. Front Physiol. 2021;12:fphys.2021.650313. 10.3389/fphys.2021.650313 PMC8181404 34108884

[bib33] Pruss KM , Marcobal A, Southwick AM et al. Mucin-derived O-glycans supplemented to diet mitigate diverse microbiota perturbations. ISME J. 2020;15:577–91. 33087860 10.1038/s41396-020-00798-6 PMC8027378

[bib34] Salli K , Hirvonen J, Siitonen J et al. Selective utilization of the human milk oligosaccharides 2′-Fucosyllactose, 3-Fucosyllactose, and difucosyllactose by various probiotic and pathogenic bacteria. J Agric Food Chem. 2021;69:182. 10.1021/acs.jafc.0c06041 33382612

[bib35] Schnorr SL , Candela M, Rampelli S et al. Gut microbiome of the Hadza hunter-gatherers. Nat Commun. 2014;5:3654. DOI: 10.1038/ncomms4654. 24736369 10.1038/ncomms4654 PMC3996546

[bib36] Sheppard A , Cammarata C, Martin DH. Comparison of different medium bases for the semiquantitative isolation of anaerobes from vaginal secretions. J Clin Microbiol. 1990;28:455–7. 2182666 10.1128/jcm.28.3.455-457.1990 PMC269643

[bib37] Stewart CJ , Ajami NJ, O'Brien JL et al. Temporal development of the gut microbiome in early childhood from the TEDDY study. Nature. 2018;562:583–8. 30356187 10.1038/s41586-018-0617-x PMC6415775

[bib38] Team RStudio . RStudio : Integrated Development for R. Boston: RStudio Inc, 2015.

[bib39] Tsukuda N , Yahagi K, Hara T et al. Key bacterial taxa and metabolic pathways affecting gut short-chain fatty acid profiles in early life. ISME J. 2021;15:2574–90. 33723382 10.1038/s41396-021-00937-7 PMC8397723

[bib40] Turck D , Castenmiller J, De Henauw S et al. Safety of lacto-N-tetraose (LNT) as a novel food pursuant to regulation (EU) 2015/2283. EFSA J. 2019;17:e05907. 32626198 10.2903/j.efsa.2019.5907 PMC7008806

[bib41] Turck D , Castenmiller J, De Henauw S et al. Safety of 6′-Sialyllactose (6′-SL) sodium salt as a novel food pursuant to regulation (EU) 2015/2283. EFSA J. 2020;18:e06097. 10.2903/j.efsa.2020.6097 PMC10464711 37649501

[bib42] Turck D , Castenmiller J, De Henauw S et al. Safety of 3-FL (3-Fucosyllactose) as a novel food pursuant to regulation (EU) 2015/2283. EFSA J. 2021;19. DOI: 10.2903/J.EFSA.2021.6662. 10.2903/j.efsa.2021.6662 PMC8243255 34221147

[bib43] Vacca M , Celano G, Calabrese FM et al. The controversial role of human gut lachnospiraceae. Microorganisms. 2020;8:573. 32326636 10.3390/microorganisms8040573 PMC7232163

[bib44] Van Den Abbeele P , Belzer C, Goossens M et al. Butyrate-producing clostridium cluster XIVa species specifically colonize mucins in an in vitro gut model. ISME J. 2013;7:949–61. 23235287 10.1038/ismej.2012.158 PMC3635240

[bib45] Vital M , Howe AC, Tiedje JM. Revealing the bacterial butyrate synthesis pathways by analyzing (meta)genomic data. MBio. 2014;5:e00889–14. 24757212 10.1128/mBio.00889-14 PMC3994512

[bib46] Walker AW , Duncan SH, Carol McWilliam Leitch E et al. pH and peptide supply can radically alter bacterial populations and short-chain fatty acid ratios within microbial communities from the human colon. Appl Environ Microbiol. 2005;71:3692–700. 16000778 10.1128/AEM.71.7.3692-3700.2005 PMC1169066

[bib47] Wang Q , Garrity GM, Tiedje JM et al. Naive Bayesian classifier for rapid assignment of rRNA sequences into the new bacterial taxonomy. Appl Environ Microbiol. 2007;73:5261–7. 17586664 10.1128/AEM.00062-07 PMC1950982

[bib48] Wrzosek L , Miquel S, Noordine ML et al. Bacteroides thetaiotaomicron and *Faecalibacterium prausnitzii* influence the production of mucus glycans and the development of goblet cells in the colonic epithelium of a gnotobiotic model rodent. BMC Biol. 2013;11:1–13. 23692866 10.1186/1741-7007-11-61 PMC3673873

[bib49] Yu ZT , Chen C, Newburg DS. Utilization of major fucosylated and sialylated human milk oligosaccharides by isolated human gut microbes. Glycobiology. 2013;23:1281–92. 24013960 10.1093/glycob/cwt065 PMC3796377

