# Supplementary material for: Human milk oligosaccharides induce acute yet reversible compositional changes in the gut microbiota of conventional mice linked to a reduction of butyrate levels
Source: Microlife. 2022 May 18;3:uqac006. doi: 10.1093/femsml/uqac006 (PMC10117735; doi:10.1093/femsml/uqac006)
Supplement: uqac006_Supplemental_Files [file uqac006_supplemental_files.zip › Supplementary_tables_all.pdf]

Table S1. Bacterial isolates recovered from intestinal samples from colon and feces.

Isolate ID	Animal No. (group)	Sample material	Sequence length (nt)	Closest BLAST hit (NCBI)	Query Cover	NCBI % Indentity	Accession	Isolate match with *Bacteroidaceae* ASV sequence[1]	Isolate % identity with ASV
FE 1	#12 (6'SL)	Feces Day 8	491	*Bacteroides caccae*	100%	99.83%	AB714287.1	YES	100%
FE 2	#12 (6'SL)	Feces Day 8	496	*Bacteroides faecichinchille*	100%	99.23%	MN854703.1	YES	100%
FE 3	#14 (6'SL)	Feces Day 8	492	*Phocaeicola vulgatus*	100%	99.03%	MN854705.1	YES	100%
FE 4	#14 (6'SL)	Feces Day 8	484	*Phocaeicola vulgatus*	100%	99.61%	MT268990.1	YES	100%
FE 5	#15 (6'SL)	Feces Day 8	495	*Phocaeicola faecalis*	100%	99.40%	MN854703.1	YES	100%
FE 6	#17 (LNT)	Feces Day 8	500	*Phocaeicola faecalis*	100%	99.29%	MN854703.1	YES	100%
FE 7	#17 (LNT)	Feces Day 8	490	*Phocaeicola faecalis*	100%	100.00%	MN854703.1	YES	100%
FE 8	#32 (3FL)	Feces Day 8	500	*Bacteroides caccae*	100%	99.22%	MK743932.1	YES	100%
FE 9	#32 (3FL)	Feces Day 8	476	*Bacteroides caccae*	100%	100.00%	AB714287.1	YES	100%
FE 10	#33 (3FL)	Feces Day 8	490	*Phocaeicola vulgatus*	100%	99.35%	MT268990.1	YES	100%
FE 11	#33 (3FL)	Feces Day 8	502	*Phocaeicola vulgatus*	100%	99.14%	MT268990.1	YES	100%
FE 12	#37 (3FL)	Feces Day 8	690	*Faecalibaculum rodentium*	100%	99.86%	CP011391.1	NO	N/A
FE 13	#37 (3FL)	Feces Day 8	744	*Faecalibaculum rodentium*	99%	99.06%	CP011391.1	NO	N/A
CO 1	#12 (6'SL)	Colon Day 8	725	*Bacteroides caecimuris*	100%	99.80%	CP015401.2	YES	100%
CO 2	#15 (6'SL)	Colon Day 8	740	*Bacteroides intestinalis*	100%	98.73%	NR_041307.1	NO	N/A
CO 3	#19 (LNT)	Colon Day 8	884	*Erysipelatoclostridium ramosum*	100%	99.55%	EU869233.1	NO	N/A
CO 4	#21 (LNT)	Colon Day 8	537	*Bacteroides caecimuris*	100%	99.23%	LC416470.1	YES	100%
CO 5	#21 (LNT)	Colon Day 8	730	*Bacteroides caecimuris*	100%	99.62%	LC416470.1	YES	100%
CO 6	#22 (LNT)	Colon Day 8	859	*Parasutterella excrementihominis*	100%	99.87%	LT558827.1	NO	N/A
CO 7	#31 (3FL)	Colon Day 8	414	*Phocaeicola faecalis*	100%	98.31%	MN854703.1	YES/NO	97.20%
CO 8	#32 (3FL)	Colon Day 8	650	*Bacteroides caccae*	100%	99.08%	AB714287.1	YES	100%

[1]The 16S rRNA gene sequence of the isolate obtained from Sanger sequencing matched an ASV sequence classified as *Bacteroidaceae* as listed in Table S2

Table S2. Prevalent amplicon sequence varients (ASVs) in 16S rRNA gene sequence data

**ASV No.**	**Read length**	**ASV % presence total samples**	**BLAST (NCBI)**	**Query Cover**	**NCBI % ID**	**NCBI Accession**	**Isolate obtained[1]**	**Species type strain**
ASV 25	155	96.59%	*Phocaeicola faecalis*	99%	98.78%	MN854705.1	YES	Not available
ASV 51	155	100%	*Phocaeicola vulgatus*	100%	100.00%	MT152628.1	NO	*Bacteroides vulgatus* (RDP : ATCC 8482)
ASV 61	155	96.59%	*Phocaeicola vulgatus*	100%	99.35%	MT515977.1	NO	*Bacteroides vulgatus* (RDP : ATCC 8482)
ASV 79	155	94.32%	*Phocaeicola vulgatus*	100%	100.00%	MT515977.1	YES	*Bacteroides vulgatus* (RDP : ATCC 8482)
ASV 121	155	98.86%	*Bacteroides caecimuris*	100%	100.00%	CP015401.1	YES	*Bacteroides caecimuris* (NCBI: CP015401)
ASV 147	155	76.14%	*Phocaeicola vulgatus*	100%	99.35%	MT152628.1	NO	*Bacteroides vulgatus* (RDP : ATCC 8482)
ASV 235	155	94.32%	*Bacteroides uniformis*	100%	100.00%	LC515590.1	NO	*Bacteroides uniformis* (RDP : AB050110)
ASV 290	155	82.95%	*Bacteroidales* sp.	100%	99.35%	LC333722.1	NO	Not available
ASV 324	154	87.50%	*Bacteroides faecichinchillae*	100%	100.00%	MK929066.1	YES	*Bacteroides faecichinchillae* (RDP : AB574480)
ASV 371	154	70.45%	*Bacteroides* sp.	100%	99.35%	MH682255.1	NO	Not available
ASV 423	155	65.91%	*Bacteroides caccae*	100%	100.00%	MT539041.1	YES	*Bacteroides caccae* (RDP : X83951)
ASV 424	155	77.27%	*Bacteroides uniformis*	100%	99.35%	LC515590.1	NO	*Bacteroides uniformis* (RDP: AB050110)
ASV 425	155	11.36%	*Bacteroides koreensis*	100%	100.00%	MT464342.1	NO	*Bacteroides ovatus* (RDP: AB050108)
ASV 851	155	20.45%	*Bacteroides fragilis*	100%	100.00%	MN629228.1	NO	*Bacteroides fragilis* (RDP: CR626927)

[1]The obtained isolate had 100% homology in terms of 16S rRNA gene sequence
