# Supplementary figures and images for: Human milk oligosaccharides induce acute yet reversible compositional changes in the gut microbiota of conventional mice linked to a reduction of butyrate levels
Source: Microlife. 2022 May 18;3:uqac006. doi: 10.1093/femsml/uqac006 (PMC10117735; doi:10.1093/femsml/uqac006)

Figure S1

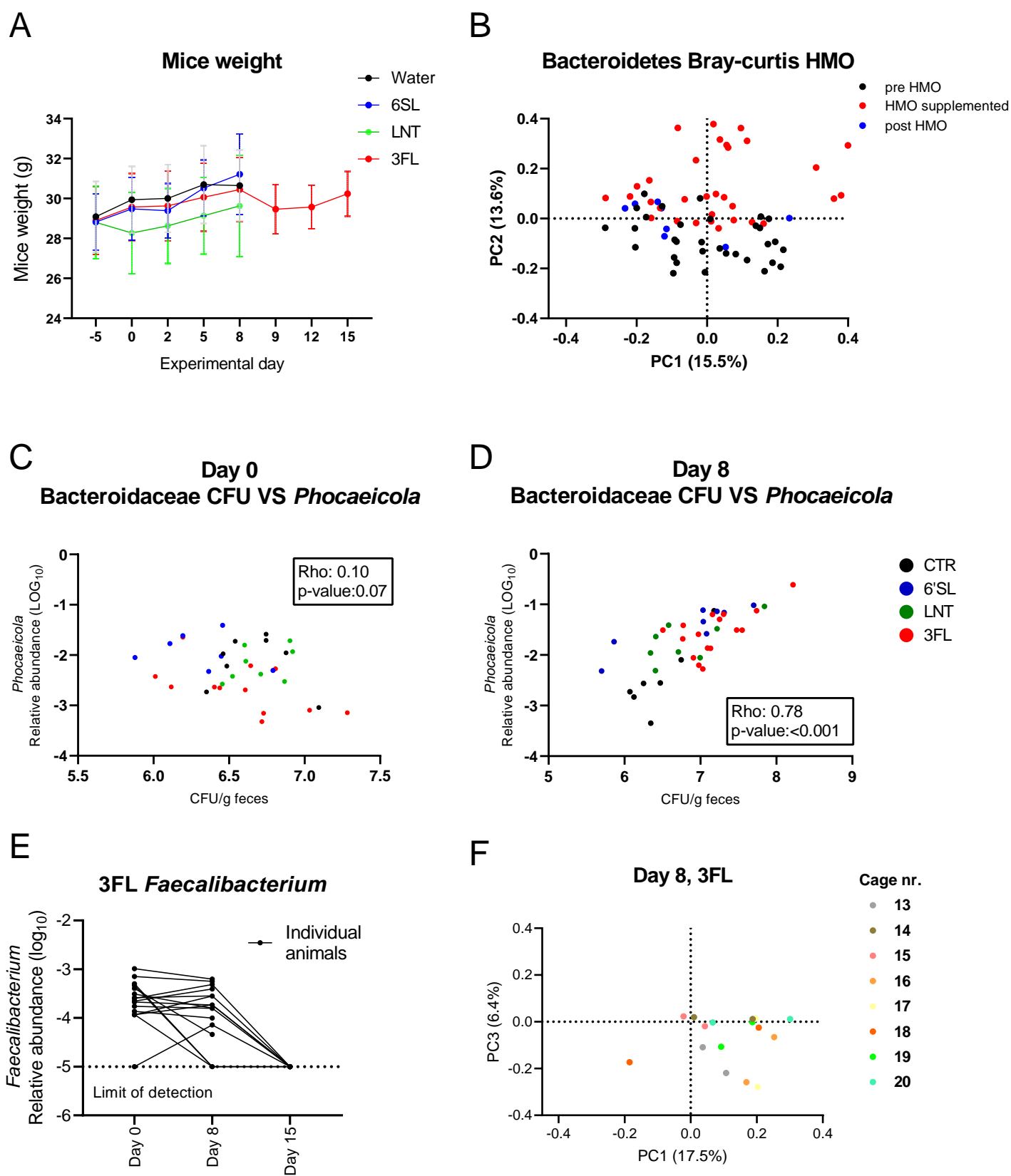

Figure S1

Figure S2

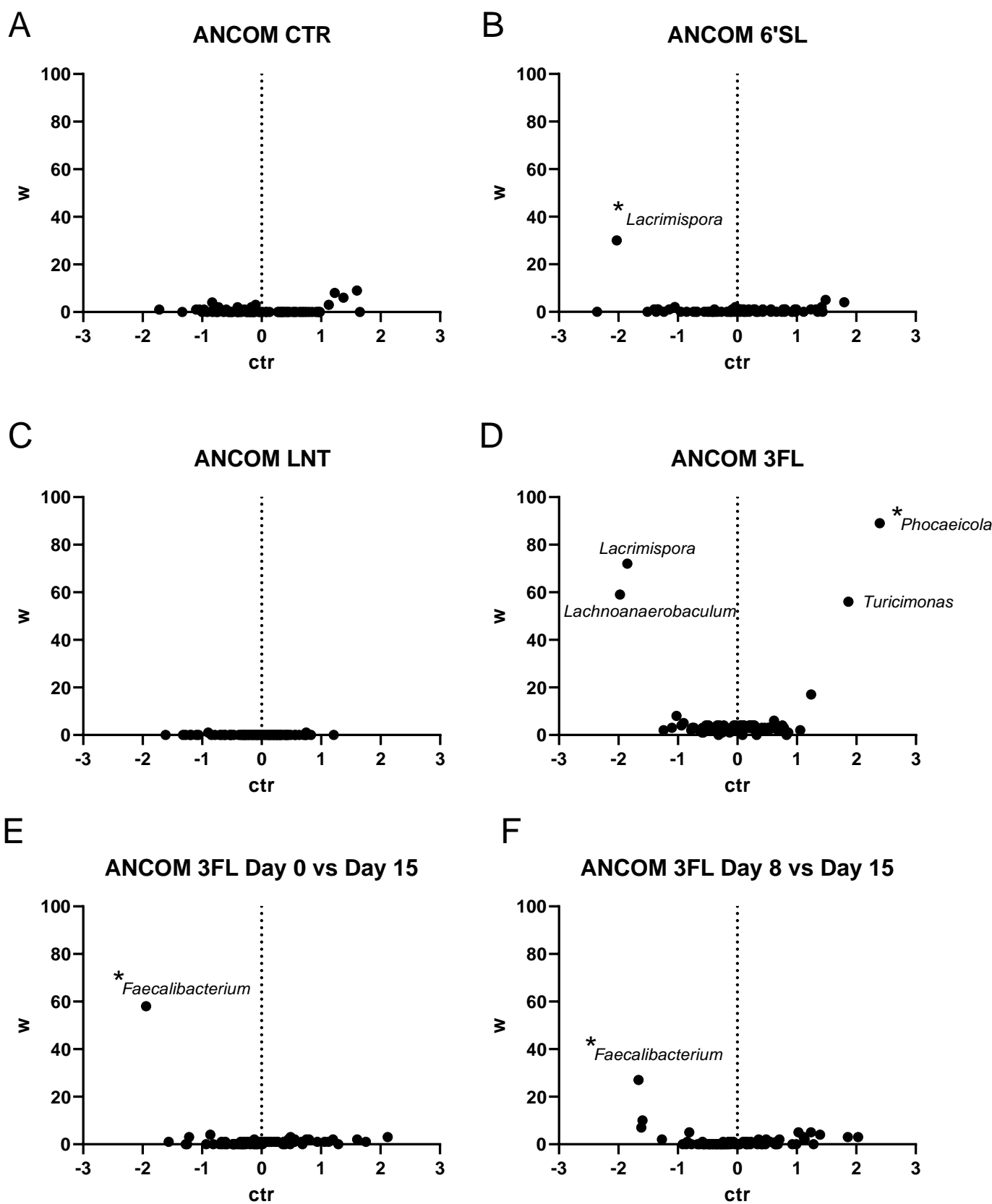

Figure S2

Figure S3

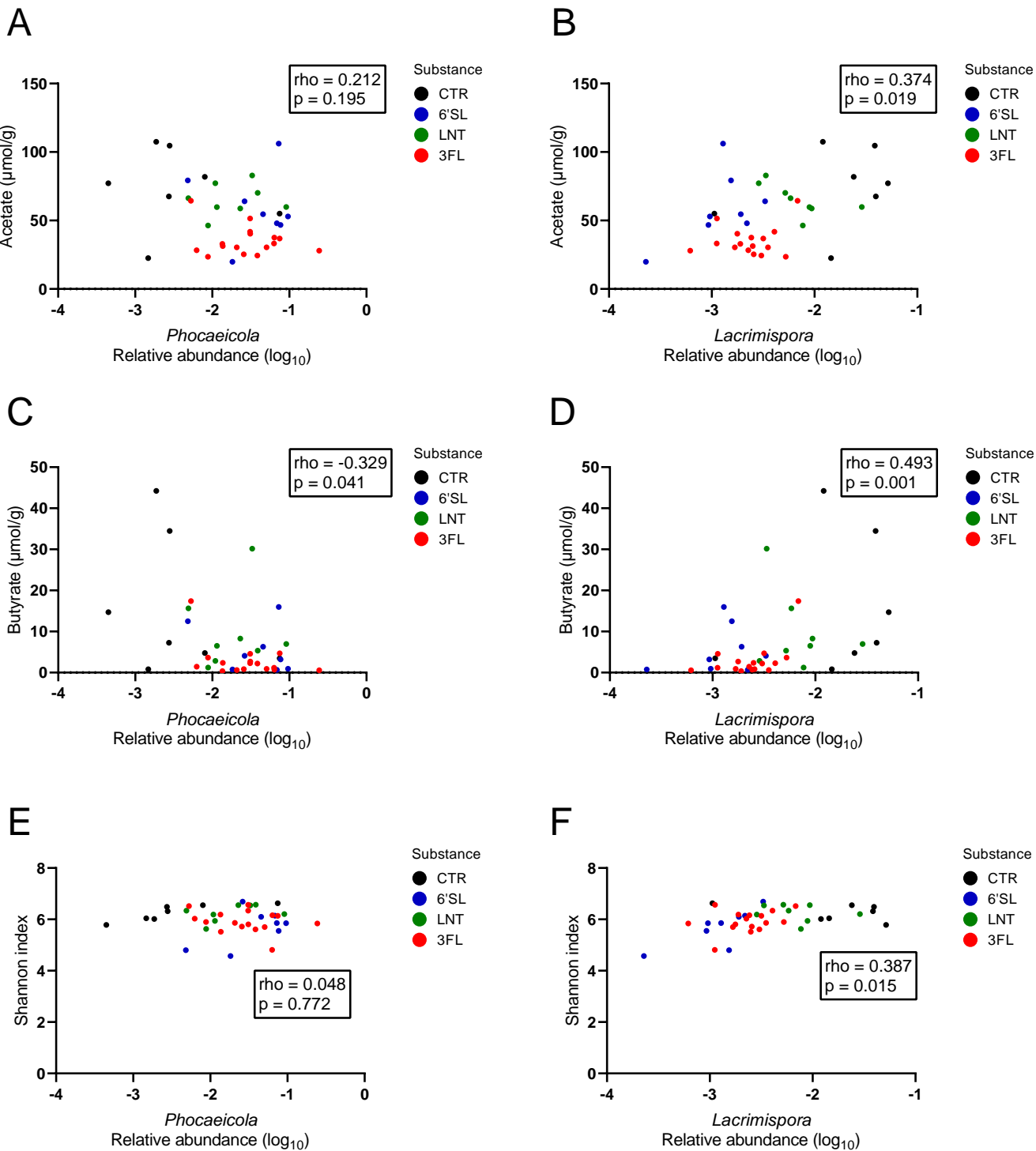

Figure S3

Supplement: uqac006_Supplemental_Files [file uqac006_supplemental_files.zip › Supplementary_figures_all.pdf]
